# Kikuchi–Fujimoto Disease Presenting With Complex Neurological Manifestations: A Case Report

**DOI:** 10.1155/crnm/9336826

**Published:** 2026-03-16

**Authors:** Yusuf Kagzi, Suban Amatya, Ashutosh Gupta, Kanika Sharma, Shitiz Sriwastava

**Affiliations:** ^1^ Department of Internal Medicine, University of Illinois College of Medicine, Peoria, Illinois, USA, uic.edu; ^2^ Department of Internal Medicine, Patan Academy of Health Sciences, Kathmandu, Nepal, pahs.edu.np; ^3^ Department of Neurology, Division of Multiple Sclerosis and Neuroimmunology, McGovern Medical School, University of Texas Health Science Center at Houston, Houston, Texas, USA, uth.edu; ^4^ Department of Neurology, College of Medicine, University of Arkansas for Medical Sciences, Little Rock, Arkansas, USA, uams.edu

**Keywords:** Kikuchi disease, neuropathy, vasculitis

## Abstract

Kikuchi–Fujimoto Disease (KFD) is a rare, typically self‐limiting inflammatory condition primarily associated with fever, cervical lymphadenopathy, and occasionally small‐vessel vasculitis. Central nervous system (CNS) involvement in KFD is exceedingly rare and has been described in isolated cases, including encephalitis, aseptic meningitis, and peripheral neuropathy. We described the case of a 43‐year‐old male of Indian descent who presented with a complex medical history of gait imbalance, falls, and slowness in daily activities, dysarthria, dysphagia, and excessive sleepiness, which had been progressively worsening over the past 16 months. His prior medical records included a diagnosis of Kikuchi disease in 2014, which manifested with fever and lymphadenopathy and resolved following steroid therapy. Subsequent evaluations ruled out other autoimmune and paraneoplastic conditions. Ultimately, a diagnosis of CNS involvement secondary to Kikuchi disease, possibly with vasculitis‐mediated pathology, was established. The patient was managed with rituximab, which significantly improved his symptoms. This case highlights the importance of considering rare and atypical neurological presentations of Kikuchi disease, along with the diagnostic challenges they pose. It is essential to conduct a thorough diagnostic workup that considers the full spectrum of disease presentations and tailors treatment to the specific underlying condition. Further, this case is an atypical and progressive presentation of KFD with extensive CNS involvement, emphasizing the role of vasculitis‐mediated pathology in such presentations and the effectiveness of rituximab in managing refractory cases.

## 1. Introduction

Kikuchi–Fujimoto Disease (KFD), also known as Kikuchi disease or histiocytic necrotizing lymphadenitis, is a rare inflammatory condition that is typically self‐limiting and primarily affects young and pediatric patients [1]. The etiology of KFD is uncertain, but it may be either infectious or autoimmune in nature. The disease has been well‐documented in systemic lupus erythematosus (SLE) and can be linked to small vessel vasculitis, manifesting as leukocytoclastic and retinal vasculitis. Central nervous system (CNS) involvement in KFD is uncommon, with only a few reported cases including encephalitis, aseptic meningitis, and peripheral neuropathy [2, 3], while some cases of KFD‐associated CNS vasculitis have responded to steroids, we present a case of steroid‐refractory KFD‐mediated CNS vasculitis that required treatment with rituximab. This case aims to highlight the diagnostic and therapeutic challenges associated with atypical KFD presentations involving the CNS.

## 2. Case Presentation

We present the case of a 43‐year‐old Indian male with progressively worsening ataxia, bradykinesia, dysarthria, dysphagia, and hypersomnolence over the past 16 months at the UT Health Neuroimmunology Clinic, University of Texas Health Science Center at Houston, USA. In 2014, he was diagnosed with biopsy‐proven Kikuchi disease due to unexplained fever and cervical lymphadenopathy, which improved within a month after steroids. In 2019, he was diagnosed with seronegative ocular myasthenia gravis, which improved with steroids and pyridostigmine. He had a history of rashes and oral ulcers.

His current symptoms started in late 2021 after an episode of pyrexia of unknown origin (PUO), associated with an insidious‐onset imbalance and swaying while walking, without vertigo or tremulousness. Notably, there was a slowness of gait but no difficulty initiating or freezing during movement. He became sluggish while performing daily activities. Although he occasionally coughed while drinking liquids, he could easily swallow solid foods. In early 2022, he developed a right middle cerebral artery (MCA) stroke with a right‐sided internal capsule infarct. He was started on dual antiplatelet therapy and recovered to baseline within weeks, with persistent fatigue. He also received a course of intravenous immunoglobulin (IVIG) therapy in the following month for relapse of his myasthenic symptoms, leading to complete remission of his symptoms. However, since late 2022, his baseline neurological symptoms have progressively worsened, including dysphagia, dysarthria, gait imbalance, and recurrent falls. His wife also reported excessive sleepiness, mild cognitive impairment, and withdrawn behavior, impacting his work life.

On examination, he exhibited bilateral structural hand and foot deformities and cervical lymphadenopathy. The patient exhibited primitive reflexes and impaired frontal lobar functions. His speech was dysarthric, with preserved comprehension and repetition. Oculomotor assessment revealed bilateral vertical gaze restriction with a slow vertical saccade and pronounced adduction restriction. There were no signs of tongue fasciculation, and cranial nerve exams were normal. The upper limbs displayed Grade 1 spasticity with normal power, and both the upper and lower limbs exhibited bilateral hyperreflexia and a positive Hoffman’s sign, along with a left‐up‐going plantar response. Sensory function remained intact; however, a cerebellar examination revealed a wide‐based gait with swaying and impaired tandem walking, without coordination deficits.

His magnetic resonance imaging (MRI) of the brain with contrast revealed multiple hyperintensities in bilateral medial temporal lobes, right insular cortex, bilateral basal ganglia, pons, and bilateral middle cerebellar peduncles with significant cerebral, midbrain, and cerebellar atrophy. There were no areas of diffusion restriction or contrast enhancement to suggest active demyelination. Bilateral enlargement of the olivary nuclei with associated signal abnormalities was consistent with hypertrophic olivary degeneration. Additional signal abnormalities were identified in the medial thalami extending to their junction with the midbrain. Further, midbrain volume loss with signal changes involving the periaqueductal gray matter was noted (Figure 1). The positron emission tomography scan with computed tomography (PET‐CT) scan of the whole body from January 2023 showed no abnormal physiological fludeoxyglucose (FDG)‐avid lesions with normal physiological FDG uptake throughout the whole body, including the brain, head and neck, chest, abdomen and pelvis, and skeletal system (Figure 1(d)). Cerebrospinal fluid (CSF) studies revealed a cell count of 11 cells/mm^3^, comprising 99% lymphocytes, normal glucose levels, elevated protein at 80 mg/dL, the presence of oligoclonal bands (OCBs), and an immunoglobulin G (IgG) index of 0.55. Due to the previous history of a right MCA stroke and the evidence of inflammation in the CSF and MRI, a comprehensive workup for vasculitis was done, which was negative for multiple autoantibodies, including antinuclear antibody (ANA), antidouble‐stranded DNA (anti‐dsDNA), perinuclear‐staining antineutrophil cytoplasmic antibody (*p*‐ANCA), cytoplasmic ANCA (c‐ANCA), proteinase 3 (PR3), myeloperoxidase (MPO), anti‐beta‐2 glycoprotein, lupus anticoagulant, anticardiolipin antibody, and antiphospholipid antibody (APLA) (Supporting Table 1). CSF analysis revealed no evidence of infectious etiology. Extensive paraneoplastic workup involving tumor markers, an abdominal ultrasound, and autoantibody paraneoplastic panel testing was also normal. Moreover, nonspecific MRI lesions and lack of CSF‐specific OCBs, as well as negative serum and CSF levels of anti‐MOG and aquaporin‐4 antibodies (AQP4), effectively ruled out demyelinating diseases such as MS, MOGAD, and NMOSD, respectively. The myasthenia panel was reassessed with antiacetylcholine receptor antibody (AChR), antimuscle‐specific kinase antibody (MUSK), and antititin antibody levels, as well as repetitive nerve stimulation, all of which displayed normal results. Additional autoimmune encephalitis screening for anti‐IgLON5, anti‐GQ1b, anti‐thyroglobulin, and antithyroperoxidase antibodies was negative. Furthermore, a skin pathergy test did not demonstrate erythematous lesions, and the human leukocyte antigen (HLA)‐B51 assay for Behçet’s syndrome was negative. Considering the patient’s multifocal CNS manifestations, including a stroke after PUO, and cervical lymphadenopathy, a diagnosis of CNS involvement secondary to Kikuchi disease, possibly with vasculitis‐mediated pathology, was established, considering his history of Kikuchi disease. The patient was initially managed with methylprednisolone 1 mg/kg for 3 years, followed by IVIG 1 gm/kg once every 4 weeks, but no improvement was observed. Subsequently, he was started on Rituximab infusions every 6 months, resulting in significant improvement in balance, posture, ocular movements, and sleep quality. Dysarthria and dysphagia partially improved, while cognitive symptoms stabilized. No further ischemic events or disease progression were noted.

FIGURE 1(a)–(c) MRI of the brain with contrast showing multiple hyperintensities involving the bilateral medial temporal lobes, right insular cortex, bilateral basal ganglia, pons, and middle cerebellar peduncles with significant cerebral, midbrain, and cerebellar atrophy. No diffusion restriction or contrast enhancement was noted, ruling out active demyelination. Bilateral olivary nucleus enlargement with signal changes consistent with hypertrophic olivary degeneration. (d) PET‐CT showing no abnormal FDG‐avid lesions, with normal physiological FDG uptake.

**Figure:**
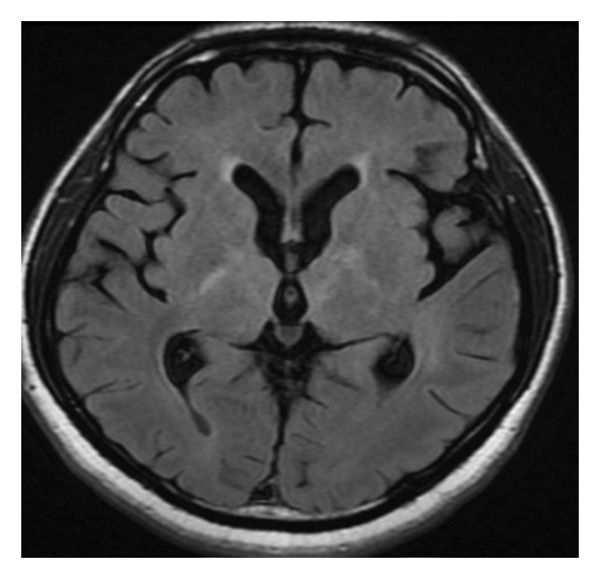
(a)

**Figure:**
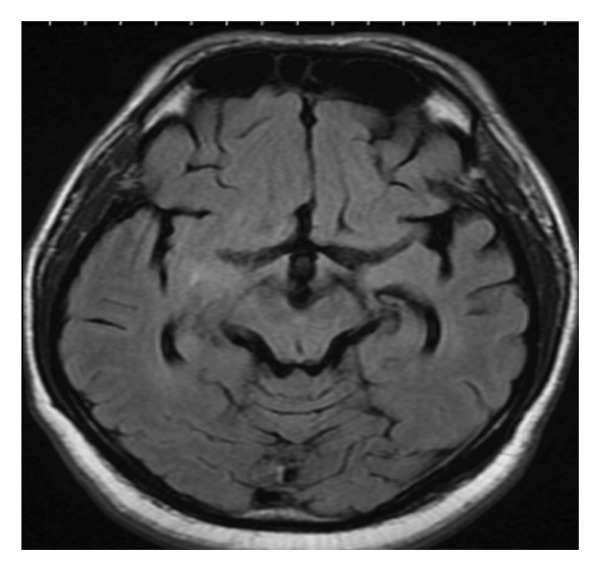
(b)

**Figure:**
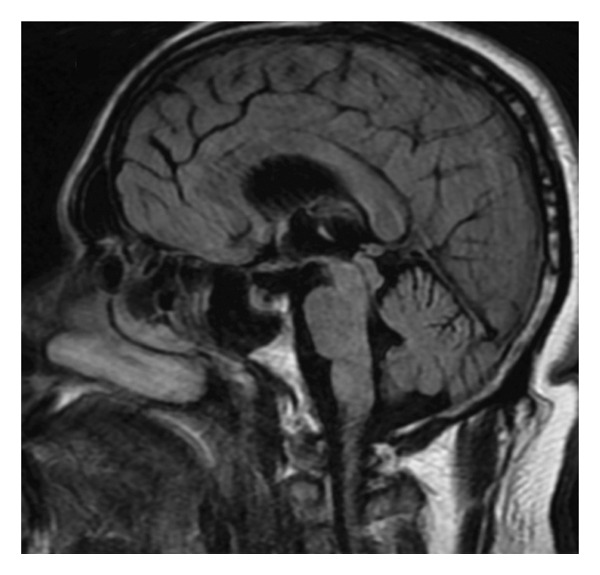
(c)

**Figure:**
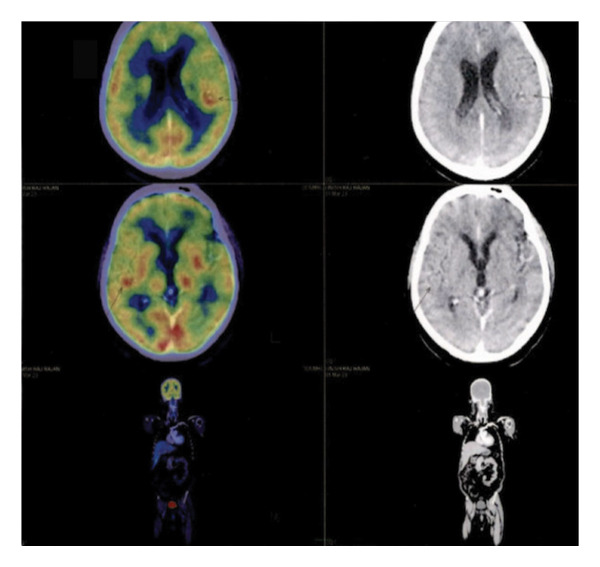
(d)

## 3. Discussion

Our patient was diagnosed with CNS involvement secondary to Kikuchi disease, considering his neurological presentation following a PUO and cervical lymphadenopathy on examination, after a diagnosis of exclusion. His diverse neurological presentation, including a lacunar stroke in MCA territory, inflammatory findings in CSF and MRI, and a positive response to rituximab, strongly suggested a vasculitis‐mediated pathology.

KFD is a rare, typically self‐limiting lymphoproliferative condition characterized by the subacute onset of fever, arthralgia, rash, and posterior cervical lymphadenopathy [1]. KFD is often associated with autoimmune conditions, primarily SLE, small vessel vasculitis, and retinal vasculitis. This small vessel vasculitis association of KFD is also manifested in our patient with his lacunar stroke and inflammatory CSF and MRI findings. While the exact etiology is unknown, many features of KFD closely resemble those of autoimmune diseases, and the positive response to immunosuppressive treatment in most cases suggests an autoimmune origin [1–3]. Rare neurologic complications of KFD have been described, including aseptic meningitis, encephalitis, and other inflammatory CNS processes, highlighting the broad spectrum of CNS involvement and supporting the plausibility of immune‐mediated mechanisms in our patient’s presentation [4].

Previous reports have documented diverse neurological manifestations of KFD, including focal weakness, meningoencephalitis, brainstem encephalitis, cerebellar ataxia, and cranial neuropathies. For instance, Goh et al. described a case of intracranial large‐vessel vasculitis with recurrent cranial neuropathies. Byun et al. reported meningoencephalitis in children with Kikuchi necrotizing lymphadenitis. Jasti et al. presented brainstem encephalitis with secondary blepharospasm, and Moon et al. detailed cerebellar ataxia as the initial manifestation. Reported MRI findings in such cases have included diffuse leptomeningeal enhancement, hemosiderin deposition, and multifocal enhancing lesions involving regions such as the temporal and occipital lobes, brainstem, cerebellar peduncles, basal ganglia, and internal capsule. Some studies have also noted perivascular and periaqueductal enhancement, as well as partial diffusion restriction [5–8]. In contrast, our patient exhibited a chronic, progressive, and steroid‐refractory neurological course spanning over 16 months, multifocal CNS involvement, and radiological evidence consistent with vasculitis. Hypersomnolence was likely related to inflammatory involvement of thalamic and midbrain structures integral to arousal pathways, rather than cortical pathology.

Previous studies have reported MRI findings, including diffuse leptomeningeal enhancement, hemosiderin deposition, and enhancing lesions in multiple brain regions (e.g., temporal and occipital lobes, brainstem, cerebellar peduncle, basal ganglia, and internal capsule). Some studies also report perivascular and periaqueductal enhancement, as well as partial diffusion restriction, as observed in our patient (Figure 1). Such patterns align with previously described chronic vasculitic pathology but represent a more advanced disease spectrum. A definitive diagnosis requires an excisional lymph node biopsy showing characteristic histopathological findings such as patchy necrotic foci, karyorrhectic debris, and surrounding histiocytic proliferation with foamy cytoplasm, i.e., histiocytic necrotizing lymphadenitis (as seen in our patient) [5–8].

A CNS or systemic biopsy was considered; however, it was not pursued due to the absence of a safe and accessible biopsy target. Moreover, the CNS lesions were multifocal and predominantly deep‐seated, involving the brainstem, basal ganglia, and thalami, and lacked focal contrast enhancement, rendering stereotactic biopsy high‐risk with low anticipated diagnostic yield. Digital subtraction angiography was likewise not performed, as it has limited sensitivity for detecting small‐vessel CNS vasculitis, which was suspected in this case. Given the invasiveness of the procedure and the anticipated low diagnostic yield, angiography was deferred. High‐resolution MR vessel wall imaging, which may have provided additional supportive evidence, was not routinely available at our institution at the time of evaluation and therefore represents a limitation of this case. While this modality may have provided supportive evidence of vasculopathy, its absence does not exclude small‐vessel inflammatory pathology. Ultimately, the diagnosis was based on the patient’s prior biopsy‐proven disease in the setting of inflammatory CSF, multifocal neurological involvement, and clinical progression [2, 8].

Rare neurologic complications of KFD have been described, including aseptic meningitis, encephalitis, and other inflammatory CNS processes. These cases highlight the spectrum of possible CNS involvement and support the plausibility of immune‐mediated mechanisms in our patient’s presentation. While most cases of KFD resolve spontaneously, some patients require additional treatment with steroids or immunosuppressants such as hydroxychloroquine [2–6]. In our case, the patient presented an atypical, severe, chronic, and progressive disease course, featuring widespread CNS involvement. Despite receiving intravenous steroids for 3 years, followed by 1 year of treatment with IVIG at a dose of 1 gm/kg, the patient reported no symptomatic relief. This presentation was likely indicative of an underlying vasculitic process secondary to KFD, necessitating robust immunosuppressive therapy with rituximab. Nonetheless, early diagnosis of KFD, primarily based on histopathology, is crucial for guiding treatment strategies and improving patient outcomes. Neurologic involvement with KFD is exceedingly rare, poorly understood, and can potentially lead to permanent disability, warranting further research to elucidate its etiopathogenesis.

## Author Contributions

Yusuf Kagzi: writing–original draft and initial literature review. Suban Amatya, Ashutosh Gupta, and Kanika Sharma: writing–original draft. Shitiz Sriwastava: conceptualization, and review and editing. Dr. Shitiz Sriwastava is the guarantor.

## Funding

No funding was used in this study.

## Ethics Statement

The article was exempt from ethics approval by the IRB at the University of Texas–Houston, as it was a single case report.

## Consent

Written informed consent was obtained from the patient for publication of this case report and all accompanying clinical data and images, in accordance with the journal’s policy. The patient reviewed the report for accuracy and provided consent for publication.

## Conflicts of Interest

The authors declare no conflicts of interest.

## Supporting Information

Supporting Table 1: Routine cerebrospinal fluid (CSF) analysis and blood autoimmune antibody testing performed as part of the diagnostic workup, including parameters such as cell count, protein levels, IgG index, and results from a comprehensive autoimmune panel.

## Supporting information


**Supporting Information** Additional supporting information can be found online in the Supporting Information section.

## Data Availability

Data will be provided on request.
