# Supplementary material for: Kikuchi–Fujimoto Disease Presenting With Complex Neurological Manifestations: A Case Report
Source: Case Rep Neurol Med. 2026 Mar 16;2026:9336826. doi: 10.1155/crnm/9336826 (PMC13329759; doi:10.1155/crnm/9336826)
Supplement: Supplementary file 1 — Supporting Information Additional supporting information can be found online in the Supporting Information section. [file CRNM-2026-9336826-s001.docx]

**Supplemental Table 1: Routine cerebrospinal fluid (CSF) analysis and blood autoimmune antibody testing performed as part of the diagnostic workup, including parameters such as cell count, protein levels, IgG index, and results from a comprehensive autoimmune panel.**

| **Parameter** | **Results** |
| --- | --- |
| **CSF Analysis:** |  |
| Cell Count | 11 cells/mm3 |
| Lymphocytes | 99% |
| Glucose | Normal |
| Protein | 80 mg/dl |
| IgG Index | 0.55 |
| Bacteria, Fungi, and Parasite | Negative |
| **Autoimmune Disease Panel:** |  |
| Antinuclear antibody (ANA) | Negative |
| Anti-double-stranded DNA (anti-dsDNA) | Negative |
| Perinuclear-staining antineutrophil cytoplasmic antibody (p-ANCA) | Negative |
| Cytoplasmic ANCA (c-ANCA), | Negative |
| Proteinase 3 (PR3) | Negative |
| Myeloperoxidase (MPO) | Negative |
| Anti-beta-2 glycoprotein | Negative |
| Lupus anticoagulant | Negative |
| Anticardiolipin antibody | Negative |
| Antiphospholipid antibody (APLA) | Negative |
| Anti-IgLON5 | Negative |
| Anti-GQ1b antibody | Negative |
| Anti-thyroglobulin antibody | Negative |
| Anti-thyroperoxidase antibody | Negative |
| **Myasthenia Gravis Panel:** |  |
| Anti-acetylcholine receptor antibody (AChR) | Negative |
| Anti-muscle-specific kinase antibody (MUSK) | Negative |
| Anti-titin antibody | Negative |
